# Extensive respiratory chain defects in inhibitory interneurones in patients with mitochondrial disease

**DOI:** 10.1111/nan.12238

**Published:** 2015-05-30

**Authors:** Nichola Z. Lax, John Grady, Alex Laude, Felix Chan, Philippa D. Hepplewhite, Grainne Gorman, Roger G. Whittaker, Yi Ng, Mark O. Cunningham, Doug M. Turnbull

**Affiliations:** ^1^ Wellcome Trust Centre for Mitochondrial ResearchInstitute of NeuroscienceNewcastle UniversityNewcastle upon TyneUK; ^2^ Bio‐imaging UnitNewcastle UniversityNewcastle upon TyneUK; ^3^ Institute of NeuroscienceNewcastle UniversityNewcastle upon TyneUK; ^4^ Department of Clinical NeurophysiologyRoyal Victoria InfirmaryNewcastle upon TyneUK

**Keywords:** cognition, epilepsy, interneurones, mitochondrial DNA, respiratory chain deficiency

## Abstract

**Aims:**

Mitochondrial disorders are among the most frequently inherited cause of neurological disease and arise due to mutations in mitochondrial or nuclear DNA. Currently, we do not understand the specific involvement of certain brain regions or selective neuronal vulnerability in mitochondrial disease. Recent studies suggest γ‐aminobutyric acid (GABA)‐ergic interneurones are particularly susceptible to respiratory chain dysfunction. In this neuropathological study, we assess the impact of mitochondrial DNA defects on inhibitory interneurones in patients with mitochondrial disease.

**Methods:**

Histochemical, immunohistochemical and immunofluorescent assays were performed on post‐mortem brain tissue from 10 patients and 10 age‐matched control individuals. We applied a quantitative immunofluorescent method to interrogate complex I and IV protein expression in mitochondria within GABAergic interneurone populations in the frontal, temporal and occipital cortices. We also evaluated the density of inhibitory interneurones in serial sections to determine if cell loss was occurring.

**Results:**

We observed significant, global reductions in complex I expression within GABAergic interneurones in frontal, temporal and occipital cortices in the majority of patients. While complex IV expression is more variable, there is reduced expression in patients harbouring m.8344A>G point mutations and *POLG* mutations. In addition to the severe respiratory chain deficiencies observed in remaining interneurones, quantification of GABAergic cell density showed a dramatic reduction in cell density suggesting interneurone loss.

**Conclusions:**

We propose that the combined loss of interneurones and severe respiratory deficiency in remaining interneurones contributes to impaired neuronal network oscillations and could underlie development of neurological deficits, such as cognitive impairment and epilepsy, in mitochondrial disease.

## Introduction

Mitochondrial diseases are heterogeneous disorders characterized by a wide‐ranging spectrum of clinical symptoms and fluctuating disease progression. Mitochondrial diseases are the result of primary respiratory chain dysfunction caused by either mitochondrial (mtDNA) or nuclear DNA mutations. Neurological deficits, along with neuromuscular involvement, are characteristic of mitochondrial disease, and these symptoms can have a dramatic impact on patient quality of life. Neurological features may be manifold, ranging from neural deafness, ataxia, peripheral neuropathy, migraine, seizures, stroke‐like episodes and dementia and depend on the part of the nervous system affected [1]. Though recent neuropathological studies are expanding our understanding of neural impairment and cell loss in the brains of patients with mitochondrial disease, the mechanisms contributing to specific pathological processes remain to be elucidated.

Recent electrophysiological studies suggest fast‐spiking, γ‐aminobutyric acid (GABA)‐ergic inhibitory interneurones are particularly vulnerable to disruption of complexes I and IV of the mitochondrial respiratory chain [2, 3]. This is not surprising as fast‐spiking inhibitory interneurones are thought to consume much more energy than other cell types in the brain, and this increased energy requirement originates from their ability to generate and sustain repetitive, high‐frequency action potentials that control complex neuronal network oscillations, known as gamma oscillations. Compared with pyramidal neurones that only display intermittent firing patterns, interneurones have elevated firing patterns which are required to support a greater metabolic load through maintenance of ionic homeostasis. Anatomical studies support the notion of a functional heterogeneity of interneurones with respect to metabolic demands. Interneurones contain a high density of mitochondria and are enriched with cytochrome *c* oxidase (COX) and cytochrome *c*; this is likely to reflect the importance of oxidative phosphorylation in supporting their high metabolic capacity [4, 5].

In the current study, we aim to establish the degree to which GABAergic inhibitory interneurones are affected by mitochondrial respiratory chain deficiencies and to determine the extent of interneurone loss in post‐mortem brain tissues from patients with mitochondrial disease.

## Methods

### Clinical assessment of patients

We investigated a total of 10 adult patients with genetically and clinically well‐defined mitochondrial disease who donated their brain for research purposes upon their death. These patients harboured different genetic defects including m.3243A>G mutation (*n* = 4), m.8344A>G mutation (*n* = 2), single large‐scale mtDNA deletion (*n* = 1) and autosomal recessive *POLG* mutations (*n* = 3). We retrospectively analysed the clinical course which was typically assessed using a validated rating scale, Newcastle Mitochondrial Disease Adult Rating Scale (NMDAS) [6], and were therefore able to document neurological deficits including ataxia, stroke‐like episodes, epilepsy and cognitive decline (Table 1). None of the patients included in this study died from their epilepsy (Table S1).

**Table 1:** A detailed clinical summary of all patients included in the current study including evidence of migraine, stroke‐like episodes and epilepsy

Patient	Age at death (years)	Gender	Genetic defect	Symptoms	Stroke‐like episodes	Epilepsy	Cognitive impairments	Electroencephalogram abnormalities	Neuropathology	Publications
Patient 1	60	F	m.3243A>G	MELAS; ataxia, dementia, deafness, diabetes, cardiomyopathy	+	+	+	Occasional burst of irregular theta activities in both temporal lobes	Microinfarcts affecting cerebellar cortex and 70% Purkinje cell loss. High levels of complex I deficiency in remaining neurones and vascular COX deficiency were reported.	Lax *et al*. [7, 8]
Patient 2	20	F	m.3243A>G	MELAS; ataxia, dementia, deafness	+	+	+	Not available	Numerous microinfarcts affecting cerebellar cortex; 40% of remaining Purkinje cell demonstrated complex I deficiency while COX‐deficient and mineralized blood vessels are evident; 5% of substantia nigra neurones are complex I and IV deficient.	Lax *et al*. and Reeve *et al*. [7, 8, 9]
Patient 3	45	M	m.3243A > G	MELAS, ataxia, dementia	+	+	+	Not available	Microinfarcts involving the cerebellar cortex. 59% and 26% cell loss from Purkinje cell and dentate nucleus neuronal populations. 50% and 30% of remaining Purkinje cells and dentate nucleus neurones are complex I deficient, respectively.	Lax *et al*. [7, 8]
Patient 4	36	F	m.3243A>G	MELAS; ataxia, deafness, constipation cardiomyopathy, dementia	+	+	+	Encephalopathic	Microinfarcts affecting the cerebellar cortex with 42%, 59% and 56% of inferior olivary neurones, Purkinje cells and dentate nucleus neurones are lost. Complex I and IV deficiency was detected in 40% and 10% of remaining Purkinje cell and dentate nucleus neurones, respectively. Vascular COX deficiency was detected in conjunction with extrusion of plasma proteins into the brain parenchyma.	Lax *et al*. [7, 8]
Patient 5	42	F	m.8344A>G	MERRF; ataxia, peripheral neuropathy, deafness, respiratory failure, dementia	−	+	+	Not available	High degree of neuronal cell loss from the olivo‐cerebellar pathway with 85% neurones lost from the inferior olives. High levels of complex I and moderate complex IV deficiencies detected in remaining neurones. COX‐deficient vasculature was also observed.	Lax *et al*. and Reeve *et al*. [7, 8, 9]
Patient 6	58	M	m.8344A>G	MERRF; ataxia, peripheral neuropathy, areflexia, dementia	+	+	+	Encephalopathic	Vascular COX deficiency observed in blood vessels throughout the cerebellum. Low levels of respiratory chain deficiency detected in Substantia nigra neurones.	Lax *et al*. and Reeve *et al*. [8, 9]
Patient 7	40	F	Single large‐scale mtDNA deletion	KSS, ataxia, dementia	−	−	+	Encephalopathic	Spongiform degeneration and loss of myelin proteins including myelin‐associated glycoprotein, attributed to respiratory chain deficiency affecting mature oligodendrocytes. 52%, 60% and 23% of olivary neurones, Purkinje cells and dentate nucleus neurones are lost. High levels of complex I deficiency affecting remaining neurones in dentate nucleus and Substantia nigra.	Lax *et al*. and Reeve *et al*. [7, 8, 9, 10]
Patient 8	24	F	*POLG (p.A467T and p.W748S and polymorphic variant: p.E1143G)*	Epilepsy, ataxia, CPEO, cognitive decline	+	+	+	Encephalopathic, right posterior quandrant spike/wave	Severe myelin loss from the posterior spinal funniculus. Severe neuronal cell loss from the olivo‐cerebellar pathway and high levels of complex I deficiency in remaining neurones. Severe neurone loss from Substantia nigra.	Lax *et al*. and Reeve *et al*. [7, 8, 9, 11]
Patient 9	55	M	*POLG (p.W748S and p.R1096C and polymorphic variant: p.E1143G)*	CPEO, ataxia, tremor, dementia	−	+	+	Encephalopathic, multifocal spikes	Not previously reported.	
Patient 10	79	M	*POLG (pT251I; p.P587L and p.A467T).*	CPEO, ataxia	−	−	−	Not investigated	Not previously reported.	

Limited clinical information was available for patients 2 and 3. MELAS, mitochondrial encephalopathy lactic acidosis and stroke‐like episodes. MERRF, myoclonic epilepsy ragged red fibres.

### Post‐mortem brain tissue

Brain tissues were processed for neuropathological examination as previously described [11], with one hemisphere snap‐frozen and the other fixed in formalin and paraffin‐embedded. In addition to the 10 brains from patients with mitochondrial disease, 10 age‐matched controls with no antecedent history of seizures or strokes were included to allow a direct comparison (Table S1). There were no significant differences in either the age at death (*t*‐test *P* = 0.9499) or post‐mortem interval (PMI; *t*‐test *P* = 0.5567). Ethical approval for this study was provided by the Newcastle and North Tyneside Local Research Ethics Committee.

To examine the interneuronal populations in these patients, we focussed our investigations on the following brain regions: frontal lobe (Brodmann area 9 – dorsolateral prefrontal cortex), temporal lobe (Brodmann area 21 – inferior temporal cortex) and occipital lobe (Brodmann area 17 – primary visual cortex) in 10 patients and 10 controls. Brain regions of interest (ROIs) were selected on the basis of recognized lobar predilection of pathological involvement in the different genotypes including occipital lobe which is commonly involved in patients harbouring *POLG* mutations, and temporal cortex in patients with m.3243A>G mutations while the frontal cortices are often described as relatively spared and hence used as a relative control ROI.

### Sequential COX and succinate dehydrogenase histochemistry

Sequential COX and succinate dehydrogenase (SDH) histochemistry were performed on three non‐serial frozen sections as previously described [12]. This allows visualization of COX‐deficient, SDH‐positive neurones (blue staining) and the COX‐positive neurones (brown staining). All neurones within 10 mm^2^ grey matter band were counted and classified as COX‐deficient (or COX intermediate which are defined as partially COX deficient and have a blue–grey appearance) or COX positive. From this data, the percentage COX‐deficient neurones were determined.

### Immunofluorescent identification of respiratory chain‐deficient GABAergic interneurones

As COX/SDH histochemistry only provides information about COX‐deficient SDH‐positive cells, we developed an immunofluorescent assay to specifically label GABAergic neurones using glutamic acid decarboxylase 65–67 (GAD65‐67), a commonly affected nuclear‐encoded subunit of complex I [NADH dehydrogenase (ubiquinone) 1 beta subcomplex subunit 8 (NDUFB8)], a mitochondrially encoded subunit of complex IV [COX subunit 1 (COX1)] and a marker of mitochondria (porin). This was performed on one 5 μm formalin‐fixed paraffin‐embedded (FFPE) section from frontal, temporal and occipital cortex from each individual patient and control, and a similar method has been described previously [13]. Our procedure briefly involves deparaffinization and rehydration of the sections followed by antigen retrieval by immersion in 1 mmol EDTA pH 8 in a pressure cooker for 40 min and washing in distilled water. Sections were blocked in tris‐buffered saline with 1% tween‐20 (TBST) containing 10% goat serum for 2 h at room temperature. Mouse monoclonal antibodies against NDUFB8 (Abcam UK Ab110242; diluted 1:100), COX1 (Abcam UK Ab14705; diluted 1:200), porin (Abcam UK Ab14734; diluted 1:200) and a rabbit polyclonal antibody directed against GAD65‐67 (Sigma UK G5163; diluted 1:1000) diluted in 10% goat serum made up TBST and incubated overnight at 4°C. This was followed by three washes in TBST, and then IgG subtype‐specific secondary anti‐mouse antibodies conjugated with Alexa Fluor 488, 546 and 647 (Life Technologies, UK) and a secondary anti‐rabbit Alexa Fluor 405 (Life Technologies, UK) antibody diluted 1:100 in 10% goat serum made up in TBST. These were incubated with the sections for 2 h at 4°C. Following this, sections were washed in three washes of TBST and then incubated in 0.3% Sudan Black for 10 min. This was followed by washing in distilled water and mounting in Prolong Gold (Life Technologies, UK).

### Imaging and densitometric analysis

Imaging of stained sections was performed using the epifluorescence Axio Imager M1 (Zeiss) microscope. Inhibitory interneurones were identified by GAD65‐67‐positive staining, and >40 neurones per case were imaged independent of cellular morphology or protein abundance. For densitometric analysis of the target proteins, Image j software (U. S. National Institutes of Health, Bethesda, Maryland, USA, version 1.46r) was used, and images from the four channels were imported as an image sequence. The background signal for all antibodies was determined in a no primary control section. Cells were outlined manually according to their GAD65‐67 signal. Within these defined areas, mean signal intensities of all channels were determined simultaneously using the ‘analyse stack’ function in Image J and an optical density (OD) value derived.

### Statistics

The background‐corrected OD values were not normally distributed and therefore required square root transformation to normalize the data. Linear regression of transformed NDUFB8 (NDUFB8^T^) data against transformed porin (porin^T^) data and transformed COX1 (COX1^T^) data against porin^T^ data was performed to ensure the residuals of the regression were normally distributed, and the standard error of estimate was determined. For each interneurone, the *z* score for the porin (porin^z^) was calculated, and the scores for NDUFB8 (NDUFB8^z^) and COX1 (COX1^z^) were derived by using the linear regression based on the level of porin. Finally, interneurones were classified based on standard deviation limits (for NDUFB8 or COX1: normal if *z* >−2SD, low if *z* <−2SD, deficient if *z* <−3SD and very deficient if *z* <−4SD). All statistical analyses were carried out using sas 9.3 (SAS Institute Inc., Cary, NC, USA).

### Immunohistochemical staining and two‐dimensional neurone counting

FFPE frontal, temporal and occipital tissues were acquired and stained for identification of GABAergic inhibitory interneurones (anti‐GAD65‐67; Sigma 1:000) using a polymer kit and visualization with DAB as previously described [11]. A two‐dimensional neuronal cell counting protocol was employed as previously described with slight modification [7]. Briefly, an area of at least 20 μm^2^ was outlined along a cortical ribbon encompassing cellular layers I – VI at ×2 magnification within each Brodmann area. As ischaemic‐like lesions were frequent in these patients, only unaffected areas of the cortex were quantified. Positive neurones as identified by dark brown chromogen staining within this area were counted at ×40 magnification and the neuronal cell densities (given as number per square millimeter) calculated for each region. This was performed on two sections separated by at least 30 μm. To determine percentage interneurone cell loss, the mean for each patient was divided by the mean interneurone density from all controls; this value was then multiplied by 100 to give percentage cell density of control. The percentage cell loss was then calculated by 100% cell density.

### Molecular biology to quantify mutation load in homogenate tissues

The percentage heteroplasmic mutant load was determined by pyrosequencing for specific point mutations in homogenate frozen frontal, temporal or occipital tissues as previously described [7].

## Results

### Neurological manifestations in patients

Clinical symptoms including neurological impairments are summarized where available by Table 1. In this study, all patients investigated displayed encephalopathic findings on electroencephalogram investigation. Eight of 10 patients had epilepsy which took a variety of forms including focal and generalized tonic–clonic or myoclonic seizures. The patient harbouring a single large‐scale mtDNA deletion (patient 7) did not have epilepsy which is entirely consistent with previous reports in the literature [14], and the other patient (patient 10) that did not have epilepsy harboured *POLG* mutations and had a primarily chronic progressive external opthalmoplegia (CPEO) phenotype and was elderly when he died. Stroke‐like episodes were also common and featured in six patients of which four patients harboured the m.3243A>G mutation, one patient harboured the m.8344A>G mutation (patient 6) and one harboured autosomal recessive mutations in *POLG* (patient 8). Patients showed signs of cognitive decline or dementia which are prominent features in patients with mitochondrial disease.

### COX deficiency in CNS tissues

**Figure 1:**
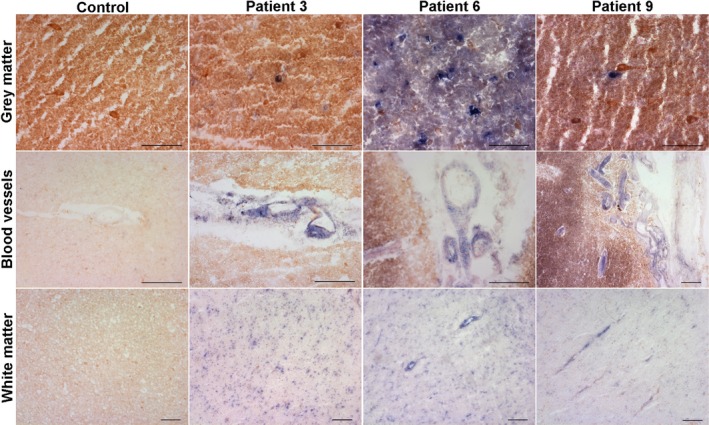
Sequential COX/SDH histochemistry reveals COX‐positive (brown) cells are present in the CNS of a control individual, while patient CNS tissues reveal a mosaic pattern of COX‐positive and COX‐deficient (blue) cells. Here, representative images have been captured from control, patient with m.3243A>G (patient 3), patient with m.8344A>G (patient 6) and patient with mutations in *POLG* (patient 9) grey matter and white matter regions in the occipital lobe. Control tissues demonstrate COX‐positive (respiratory normal) cells throughout all regions investigated, while patient tissues show both COX‐positive and COX‐deficient cells affecting neurones, blood vessels and glia. Scale bar = 100 μm.

Sequential COX/SDH histochemistry reveals the presence of COX‐deficient cells throughout the CNS (Figure 1). The long PMI for patient 2 did not appear to negatively affect either COX or SDH enzyme activity in all CNS regions assayed; though COX activity was certainly deficient in some neurones, there were multiple neurones which demonstrated high activity (strong DAB reaction product), while SDH activity was high in neurones and blood vessels that were COX‐deficient. Analysis of the frontal, temporal and occipital tissues highlights COX‐deficiency present in neurones. Quantification of the percentage of neurones harbouring COX‐deficiency revealed the highest percentage of COX‐deficient neurones in patients harbouring m.8344A>G, while other patients had COX‐deficient neurones at lower levels (summarized in Table 2). Almost all patients (including those harbouring *POLG* mutations) demonstrated COX‐deficiency within blood vessels and white matter tracts. Although this provides information about the distribution and extent of COX‐deficiency, this method is limited as it does not distinguish exactly which cells are most vulnerable and assessment of COX/SDH histochemistry is subjective.

**Table 2:** COX deficiency throughout the frontal, temporal and occipital cortices

Patient	% COX‐deficient neurones	COX‐deficient neuropil	COX‐deficient cells in white matter	COX‐deficient blood vessels
Frontal cortex				
Patient 1	0	No	No	No
Patient 2	37	Yes	Yes	Yes
Patient 3	10	No	No	Yes
Patient 5	62	Yes	Yes	Yes
Patient 6	59	Yes	*	Yes
Patient 7	2	No	Yes	Yes
Temporal cortex				
Patient 1	2	No	Yes	Yes
Patient 2	26	Yes	Yes	Yes
Patient 3	37	Yes	Yes	Yes
Patient 5	64	Yes	Yes	Yes
Patient 6	82	Yes	Yes	Yes
Patient 7	8	Yes	Yes	Yes
Occipital cortex				
Patient 3	24	Yes	Yes	Yes
Patient 5	75	Yes	Yes	Yes
Patient 6	88	Yes	Yes	Yes
Patient 7	27	Yes	Yes	Yes
Patient 8	27	Yes	Yes	Yes
Patient 9	36	Yes	Yes	Yes
Patient 10	36	Yes	Yes	Yes

*There was no white matter present on the frontal lobe section obtained for patient 6, and therefore, this could not be assessed.

### Respiratory chain deficient inhibitory interneurones

**Figure 2:**
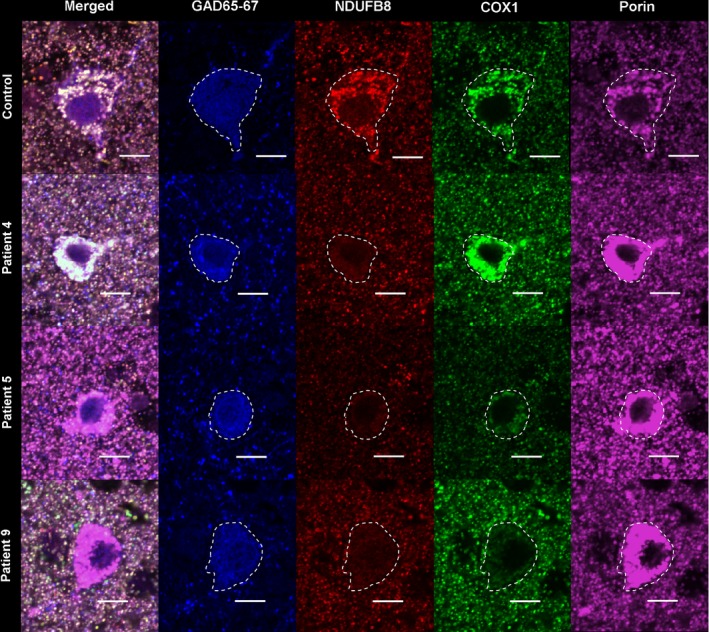
Quadruple immunofluorescence allows visualization of mitochondrial respiratory chain proteins including complexes I and IV in conjunction with mitochondrial mass within GABAergic interneurones in the inferior temporal lobe. Here, representative images have been captured of GABAergic inhibitory interneurones (GAD65‐67‐positive cells which are delineated by white dashed line) from control, m.3243A>G, m.8344A>G and *POLG* individuals in the inferior temporal cortex. Control GABAergic interneurones show equal expression of complex I (NDUFB8) and complex IV (COX1) and mitochondria (porin) which colocalize in the merged image. In m.3243A>G, there is a specific loss of NDUFB8, while COX1 and porin remain intact. Both m.8344A>G and *POLG* show a combined loss of NDUFB8 and COX1 while mitochondrial density is high. Scale bar = 10 μm.

We defined respiratory chain deficiency as a loss or reduced immunoreactivity of either NDUFB8 or COX1 proteins, despite high immunoreactivity of porin for mitochondria within a GABAergic inhibitory interneurone cell body. Throughout all cortical regions assessed, we detected a range of respiratory chain deficiencies within GABAergic interneurones by quadruple immunofluorescent staining (Figure 2). We visually confirmed that control GABAergic neurones showed co‐localization of NDUFB8, COX1 and porin implying intact mitochondrial respiratory chain protein expression in these cells. However, in patient tissues, there was an obvious lack of co‐localization due to a loss of NDUFB8 and COX1 expression despite high expression of porin in GABAergic interneurones. It was clear that the inhibitory interneurone populations exhibit respiratory chain deficiencies to varying levels in all CNS regions in all patients.

NDUFB8 expression was typically more affected than COX1 indicating more complex I deficiency though this pattern was variable. Patients harbouring the m.3243A>G mutation and single large‐scale mtDNA deletion showed a more specific loss or reduced expression of NDUFB8 while COX1 expression appears relatively intact. Patients harbouring the m.8344A>G point mutation or *POLG* mutations showed combined deficiencies of complexes I and IV (Figure 2). Loss of NDUFB8 and COX1 expression may occur with increased porin levels in patient interneurones (Figure 2).

**Figure 3:**
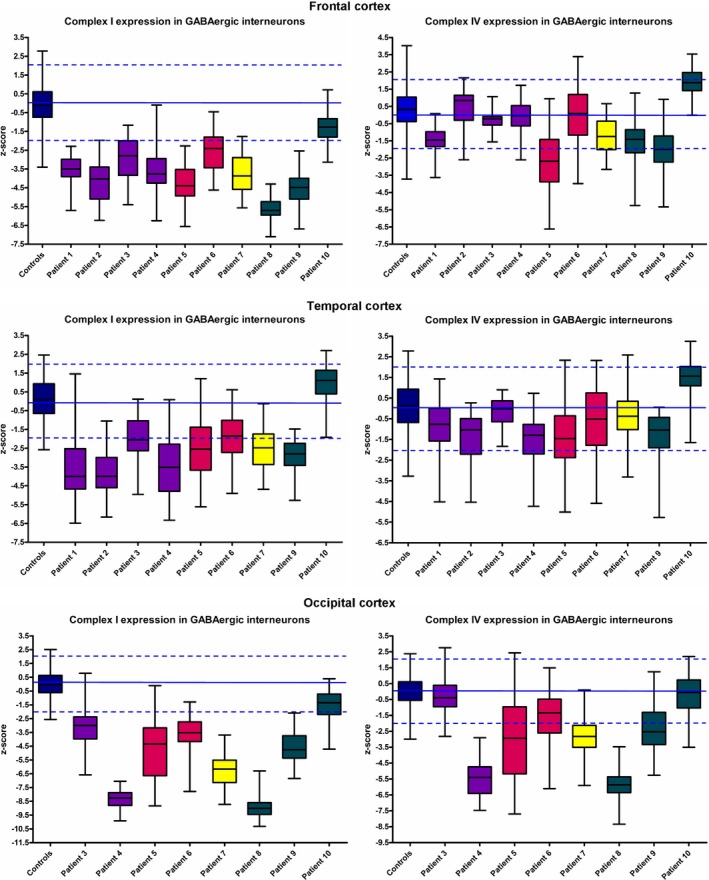
Extensive respiratory chain deficiency involving complex I and to a lesser degree complex IV is evident in GABAergic interneurones throughout the CNS in patients with mitochondrial disease. Data represents *z* scores derived from the quantitative assessment of NDUFB8 and COX1 optical densities relative to porin optical densities within GABAergic interneurones in patients and controls. The data show how much the complex I and IV expression (relative to porin) deviates from normality. Here, a *z* score of −2 to +2 indicates normal expression (dashed blue lines demonstrate upper and lower limits of normal expression), while a *z* score above 2 indicates higher expression, and a *z* score lower than −2 indicates reduced expression. There is marked complex I deficiency, while complex IV deficiency is only slight in comparison in all patient interneurones.

Quantification of OD for NDUFB8, COX1 and porin was performed within each GAD65‐67 positive neuronal cell body, and to determine respiratory chain deficiency, the OD data were transformed with linear regression for NDUFB8 *vs.* porin and COX1 *vs.* Porin, and *z* scores were derived (as shown in Figure 3). In this study, we used negative *z* scores of magnitude 3 or less to indicate respiratory chain deficiency, and based on this, we determined the percentage of interneurones exhibiting respiratory chain deficiency (Table 3).

**Table 3:** Percentage of GABAergic interneurones exhibiting complex I and IV deficiency

	Controls	Patient 1	Patient 2	Patient 3	Patient 4	Patient 5	Patient 6	Patient 7	Patient 8	Patient 9	Patient 10
Frontal cortex											
% NDUFB8 deficiency	0.3	73	89	47	75	96	36	69	100	96	2
% COX1 deficiency	0.2	5	0	0	0	45	5	3	8	17	0
Temporal cortex											
% NDUFB8 deficiency	0	69	79	18	66	40	21	33		37	0
% COX1 deficiency	0.5	4	16	0	5	15	5	2		4	0
Occipital cortex											
% NDUFB8 deficiency	0			50	100	79	68	100	100	94	9
% COX1 deficiency	0			0	98	49	17	39	100	33	4

Our quantitative data show interneurones display prominent deficiencies for complex I in almost all patients with fewer complex IV‐deficient cells throughout all three CNS regions investigated (Figure 3 and Table 3). In agreement with visual observations, interneurones sampled from patients harbouring the m.3243A>G mutation exhibit the greatest deficiency for NDUFB8, while COX1 involvement is minimal (Table 3). Quantification shows evidence of combined complex I and IV deficiency in patients harbouring m.8344A>G (patient 5) and *POLG* mutations (patient 8 and 9). Intriguingly, patient 10 (POLG) demonstrates very mild respiratory chain deficiency in interneurones with most being preserved, though it is important to note this patients' clinical course was mild and he had a CPEO phenotype, whereas patient 8 (POLG) had an early‐onset, Alpers'‐like devastating disease course.

### GABAergic interneurone cell loss in mitochondrial disease

**Figure 4:**
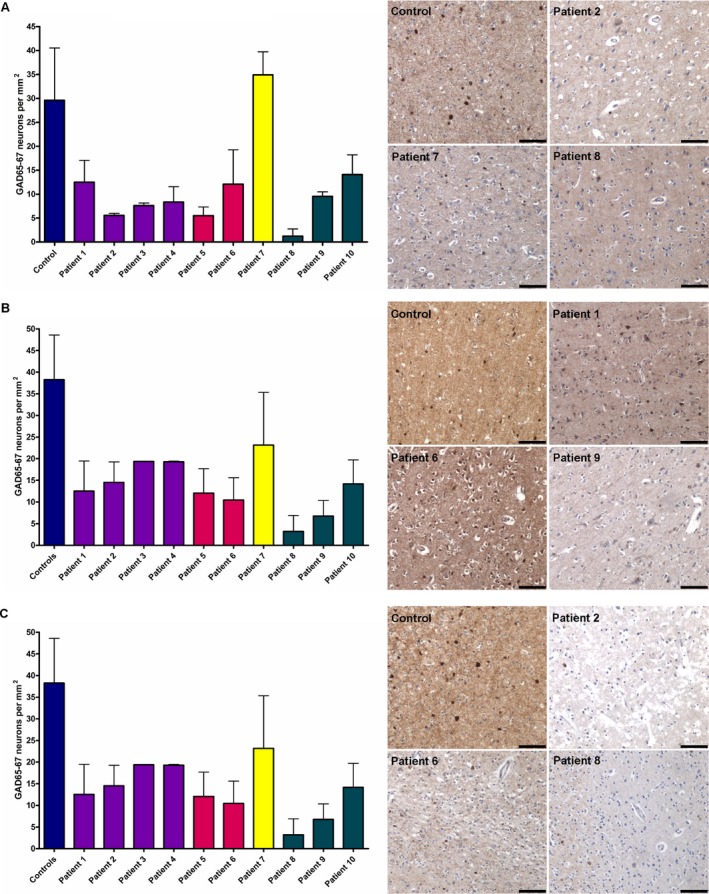
GABAergic interneurone loss is evident in frontal, temporal and occipital cortices in patients with mitochondrial disease relative to age‐matched controls. Quantification of GAD65‐67‐positive cells in frontal (**A**), temporal (**B**) and occipital (**C**) cortices are shown as bars with mean ± standard deviation. In all patients, there is a clear reduction in the density of GABAergic interneurones in all brain regions assessed. In addition to reduced number of GAD65‐67‐positive cells, there is also variability in immunoreactivity with a propensity towards reduced expression within the neurites of patients' tissues relative to controls. Scale bar = 100 μm.

We demonstrated that GABAergic cell density is reduced throughout all cortical regions assessed in the majority of patients relative to their control counterparts (Figure 4). Quantification of GAD65‐67‐positive cells and therefore GABAergic cell density revealed a reduction in all regions examined suggesting widespread and, in some patients, a high percentage (>50%) loss of GABAergic cells throughout the CNS (Figure 4 and Table 4). This analysis did not reveal that any particular CNS region was more vulnerable than another. There is also variability in the immunoreactivity for GAD65‐67 with the majority of patients revealing a loss of positive staining in neuritic processes which implies that inhibitory neurotransmission may be affected. We evaluated whether there was a correlation between respiratory chain deficiency involving complex I and complex IV and the percentage of interneurone loss. Though there was a positive trend with increasing cell loss and increased respiratory chain deficiency in remaining cells, this did not reach statistical significance (Spearman's rank correlation coefficient).

**Table 4:** Percentage of GABAergic neurone cell loss in patients (patient neurone density/average control neurone density × 100)

Patient	Frontal cortex (%)	Temporal cortex (%)	Occipital cortex (%)
Patient 1	58	67	Not available
Patient 2	81	62	Not available*
Patient 3	74	49	67
Patient 4	72	50	87
Patient 5	81	68	70
Patient 6	59	73	67
Patient 7	0	39	44
Patient 8	96	92	81
Patient 9	68	82	88
Patient 10	52	63	60

*Data for patient 2 are not available as the occipital cortex was completely devastated by a necrotic ischaemic‐like lesion.

### Percentage mutant load in frontal, temporal and occipital homogenates

Percentage m.3243A>G and m.8344A>G mutation loads determined in homogenate tissues are shown in Table 5. Molecular analysis showed that the level of mutation did not vary across the three brain regions and was greater than 79% in all patients analysed.

**Table 5:** Percentage mutant load in homogenate CNS tissues

Patient	Frontal cortex	Temporal cortex	Occipital cortex
Patient 1	79	83	Not available
Patient 2	93	93	94
Patient 3	90	94	92
Patient 5	92	92	93
Patient 6	87	88	88

## Discussion

We investigated the neuropathology of 10 adult patients with mitochondrial disease to determine whether inhibitory interneurones are particularly vulnerable to mtDNA defects and could feasibly contribute to altered neuronal network circuitry in the brain. Using a quadruple immunofluorescent technique, we provide clear evidence of combined respiratory chain deficiencies involving complexes I and IV, despite high mitochondrial mass, in GABAergic interneurones throughout the CNS of patients with mitochondrial disease. The overall density of GABAergic interneurones is reduced in many of these patients, implying reduced inhibitory neurotransmission which could contribute to altered network dynamics in the brains of patients. This raises the possibility that a loss of inhibitory interneurones might lower the amount of inhibitory activity in the brain and could contribute to phenotypes, including epilepsy and cognitive decline.

Recent electrophysiological evidence implicates a vulnerability of fast‐spiking inhibitory interneurones to perturbed energy metabolism [2, 3, 15]. The evidence for this vulnerability stems from the high energy requirement of these cells to produce complex, high‐frequency action potentials allowing them to exert local control over large populations of pyramidal neurones. Inhibitory GABAergic interneurones modulate excitation and inhibition in the cerebral cortex [16] and thereby contribute to local control of microcircuitry, shaping information outputs from the excitatory pyramidal neurones which transmit information throughout the brain to different cortical areas. The balance of excitation and inhibition is crucial, and without this, the threshold for developing pathological conditions, such as epilepsy or impaired cognition, might be lowered. Indeed, dysfunction or a loss of GABAergic interneurones has been recognized as a potential contributor to lowering seizure threshold [17, 18, 19]. This work shows that there is indeed widespread mitochondrial respiratory chain deficiency in inhibitory interneurones in patients with mitochondrial disease, and this might contribute to altered network dynamics in the brains of these patients.

In this study, we used a quadruple immunofluorescent approach to accurately quantify respiratory chain deficiency by measuring complex I and IV subunit expression in conjunction with mitochondrial mass in GABAergic interneurone populations in patients with mitochondrial disease. This method provides an unbiased, reproducible, precise and detailed analysis of mitochondrial respiratory chain deficiency which can be modified to include other subunits of the mitochondrial respiratory chain or used to evaluated different cell types. Our data reveal extensive respiratory chain deficiencies affecting inhibitory interneurones, with a marked reduction in complex I and IV expression in patients with mitochondrial disease relative to age‐matched controls. Complex I deficiency exceeds complex IV deficiency which is a consistently reported neuropathological finding in patients with mitochondrial disease [20, 21] and is a common pathological finding in other neurological disorders, including Parkinson's disease [22] and temporal lobe epilepsy [23].

We next investigated interneurone population density to determine the impact of respiratory chain deficiency on interneurone cell loss. We observed a widespread and extensive decrease in interneurone density throughout all brain regions analysed in the majority of patients. An exception to this is patient 7 (single mtDNA deletion) where the density of interneurone was similar to control densities in frontal cortex, and higher than in other patients in temporal and occipital cortices. All quantification was performed in areas of the cortex that were spared and did not show features consistent with laminar dehiscence or necrotic lesions. What is interesting is the trend towards interneurone loss, affecting all regions including the frontal cortex. Pathology in these patients is often described in posterior regions [12, 24], though in our clinical experience, frontal lobe involvement is not uncommon. In fact, ischaemic‐like lesions in frontal lobes were documented in four patients in this study, including patient 1 (m.3243A>G), patient 2 (m.3243A>G), patient 9 (*POLG*) and patient 10 (*POLG*) which suggests greater brain involvement than perhaps originally perceived [25, 26]. Indeed, high heteroplasmy levels of mutated mtDNA were detected in homogenate DNA extracted from all three brain regions. Interneurone pathology in these frontal regions may also influence other pathological processes recognized in mitochondrial disease, including neuropsychiatric manifestations and cognitive decline [27]. Indeed, cognitive decline has been reported in patients with mitochondrial disease previously [28, 29], and a loss of calbindin‐positive interneurones is implicated in the development of cognitive impairments in patients harbouring the m.3243A>G mutation [30]. Interneurone cell loss was extensive in the majority of patients but was less severe in patient 7 (single large‐scale deletion) and patient 10 (POLG) where GABAergic interneurones were only moderately decreased. Pathology in patients harbouring large‐scale mtDNA deletions predominantly results from spongiform degeneration of the white matter tracts and is associated with cerebellar ataxia and cognitive impairment [14, 31]. Patient 10 (POLG) presented with mitochondrial disease later in life and principally exhibited a CPEO phenotype without overt neurological involvement, and this may account for why there was very little respiratory chain deficiency in his interneurones and a moderate loss of interneurones.

Our data show extensive involvement of the interneurones in mitochondrial disease, and we believe that interneurone pathology due to respiratory chain deficiency and cell loss might contribute to altered neuronal network dynamics in the brain which is responsible for neurological deficits seen in these patients. Electroencephalogram data from these patients certainly provides evidence in support of impaired neuronal networks in mitochondrial disease, and we believe that interneurone dysfunction and cell loss could be one factor contributing to abnormal neuronal activity. In those patients where interneurone loss is high it is interesting to note that the clinical course was generally dominated by severe neurological impairments including stroke‐like episodes, seizures and cognitive decline. Although other factors, such as mitochondrial dysfunction in other cell types, may contribute and certainly may have an additive effect on development and severity of neurological disease. Certainly, COX/SDH histochemistry reveals that COX‐deficiency affects multiple cell types including neurones, vascular and glial cells (Figure 1). The contribution of mitochondrial impairments in these cells may modify neuronal network oscillations and may be an important factor for the development of multiple neurological features, including stroke‐like episodes and epilepsy [32]. Therefore, it would be important to assess the degree of mitochondrial impairment in these cells.

Though neuropathological investigation of post‐mortem brain tissue from patients with mitochondrial disease is limited as pathology often represents end‐stage disease and patients with severe end‐stage disease are much more likely to have had severe neurological conditions, we believe this study has provided clear evidence of mitochondrial dysfunction in GABAergic interneurones. We believe interneurone dysfunction is an important finding and might be one of the pathological processes contributing to altered neuronal oscillations to promote development of impaired neurological function in these patients. This work offers an important platform from which further work can be completed to understand how mitochondrial respiratory chain deficiencies affect distinct neuronal populations.

## Conflict of interest

The authors declare that they have no conflict of interest.

## Author contributions

NZL, MOC and DMT contributed substantially to the conception of the study. NZL, AL and PDH contributed to the design of the study. GG, RGW and YN acquired the clinical information. NZL acquired the data. NZL and JP performed the data analysis. NZL, JP, GG and DMT contributed to data interpretation, and all authors provided critical revisions and gave their final approval to the manuscript.

## Supporting information

Table S1. Characteristics of the patient and control tissue used for this study.
