# Supplementary material for: Extensive respiratory chain defects in inhibitory interneurones in patients with mitochondrial disease
Source: Neuropathol Appl Neurobiol. 2015 May 30;42(2):180–93. doi: 10.1111/nan.12238 (PMC4772453; doi:10.1111/nan.12238)
Supplement: Supplementary file 1 — Table S1. Characteristics of the patient and control tissue used for this study. [file NAN-42-180-s001.docx]

**Supplementary information**

Control details

| **Case ID** | **Category** | **Age** | **PMI** | **Gender** | **Cause of death** |
| --- | --- | --- | --- | --- | --- |
| Control 1 | Control | 48 | 72 | M | Coronary artery atherosclerosis |
| Control 2 | Control | 48 | 46 | M | Coronary artery atherosclerosis |
| Control 3 | Control | 61 | 61 | M | Hanging |
| Control 4 | Control | 48 | 43 | M | Coronary artery thrombosis |
| Control 5 | Control | 25 | 53 | M | Hanging |
| Control 6 | Control | 44 | 83 | M | Complications of bronchopneumonia and Coronary artery atherosclerosis |
| Control 7 | Control | 44 | 70 | F | Unascertained |
| Control 8 | Control | 45 | 93 | F | Coronary artery atherosclerosis |
| Control 9 | Control | 45 | 83 | M | Coronary artery thrombosis |
| Control 10 | Control | 47 | 42 | M | Coronary artery atherosclerosis |

Patient details

| **Case ID** | **Category** | **Age** | **PMI** | **Gender** | **Cause of death** |
| --- | --- | --- | --- | --- | --- |
| Patient 1 | m.3243A>G | 60 | 10 | F | Cardiorespiratory failure |
| Patient 2 | m.3243A>G | 20 | 187 | F | Aspiration Pneumonia and MELAS |
| Patient 3 | m.3243A>G | 45 | 43 | M | Gastrointestinal bleed |
| Patient 4 | m.3243A>G | 36 | 42 | F | Cardiorespiratory failure |
| Patient 5 | m.8344A>G | 42 | 59 | F | Respiratory failure |
| Patient 6 | m.8344A>G | 58 | 66 | M | Stroke-like episode |
| Patient 7 | Single large scale deletion | 40 | 58 | F | Encephalopathy |
| Patient 8 | *POLG* | 24 | 83 | F | Suppurative tracheobronchitis |
| Patient 9 | *POLG* | 55 | 112 | M | Respiratory failure |
| Patient 10 | *POLG* | 79 | 85 | M | Respiratory failure |

**Supplementary Table 1 – Characteristics of the patient and control tissue used for this study.**
